# Clinicopathological analysis of CD8-positive lymphocytes in the tumor parenchyma and stroma of hepatocellular carcinoma

**DOI:** 10.3892/ol.2014.2516

**Published:** 2014-09-09

**Authors:** JUN-LING AN, QIAO-HONG JI, JI-JIANG AN, SHINJI MASUDA, KOICHI TSUNEYAMA

**Affiliations:** 1 Department of Pathology and Pathophysiology, Medical College, Henan University of Science and Technology, Luoyang, Henan 471003, P.R. China; 2 The Emergency Department, Songxian People’s Hospital, Luoyang, Henan 471400, P.R. China; 3 Department of Diagnostic Pathology, Koseiren Takaoka Hospital, Toyama 933-0335, Japan; 4 Department of Diagnostic Pathology, Graduate School of Medicine and Pharmaceutical Sciences, University of Toyama, Toyama 930-0194, Japan

**Keywords:** hepatocellular carcinoma, tumor parenchyma, tumor stroma, CD4 T cells, CD8 T cells

## Abstract

Tumor-infiltrating lymphocytes may be a manifestation of antitumor immunity. In the present study, hepatocellular carcinoma (HCC) and pericancerous non-tumor liver tissues samples were obtained from 86 surgical patients who had not received preoperative treatment. The cellular expression levels of CD4 and CD8 were immunohistochemically examined in the two tissue groups using tissue microarrays, to evaluate their clinicopathological relevance. Immunohistochemically, CD4 and CD8 T cells were observed in the tumor parenchyma and tumor stroma, and the intensity of CD4 and CD8 immunoreactivity was homogeneous in all HCC samples examined. Morphometrically, the average numbers of CD4- and CD8-positive T cells were significantly increased in the tumor stroma, compared with those in the tumor parenchyma (tumor stroma versus tumor parenchyma: 22±3.6 versus 7.4±0.9 in CD4, 32.8±4.2 versus 16±2.5 in CD8; both P<0.01). Furthermore, the average numbers of CD8-positive T cells in the tumor parenchyma and stroma were significantly increased, compared with the average numbers of CD4-positive cells (P<0.05). In addition, in the tumor parenchyma and stroma, the average numbers of CD8 T cells were significantly higher in patients with tumor diameters ≤5 cm compared with those in patients with tumor diameters >5 cm (diameter ≤5 cm versus diameter >5 cm: 18.1±3.3 versus 12.2±3.8 in tumor parenchyma, 36.5±4.8 versus 21.9±8.9 in tumor stroma; both P<0.05). In addition, CD8 expression was significantly enhanced in patients with chronic hepatitis and cirrhosis, compared with paired tumor parenchymal tissues (P<0.01). Furthermore, a significant positive correlation was observed between CD4 and CD8 expression in the tumor parenchyma and stroma (both P<0.001). These observations suggest that tumor parenchyma- or stroma-infiltrating CD8 T cells may be involved in HCC tumor diameter control.

## Introduction

Hepatocellular carcinoma (HCC) is the fifth most common type of cancer, with >600,000 HCC cases developing annually worldwide (1). HCC commonly occurs in patients secondary to chronic hepatitis or cirrhosis resulting from either hepatitis B or C virus infection, or from non-virus-related causes, such as alcohol or aflatoxin exposure (2–4). A persistent, non-specific and ineffective immune system activation within the chronically inflamed liver is hypothesized to induce carcinogenesis (2,3,5). Current treatments for HCC include surgical resection, liver transplantation and local ablative therapies, such as percutaneous ethanol injection, thermal ablation and intra-arterial chemoembolization (6). However, >75% patients relapse within five years and the overall survival for HCC patients remains poor (7,8). Therefore, the development of more effective therapeutic tools and strategies is required.

A number of studies have suggested that the tumor microenvironment is important in tumor development, tumor control and the response to treatment (9–14). In breast, colorectal and lung cancer, as well as HCC, the status of the stroma and the local adaptive immune response are superior prognostic factors compared with tumor phenotype or clinical staging (11–14). In clinicopathological practice, intratumoral infiltration of CD4 or CD8 T cells was found to be correlated with lower disease recurrence and improved survival rates in HCC (14–16) and ovarian carcinoma (17). Furthermore, HCC tumor size was also found to have prognostic significance (7,18,19). In human colorectal tumors, the type, density and location of tumor-infiltrating immune cells have been reported to be predictors of clinical outcome (11). In a transgenic mice model, the adoptive transfer of CD8-positive cytotoxic T lymphocytes (CTLs) into immune-deficient mice markedly reduced tumor growth and tumor diameter (20). However, in human HCC, the association between tumor-infiltrating immune cells and tumor size is less understood. In the present study, the association between T-cell type, location and the biological behavior in human HCC specimens was investigated, particularly focusing on CD4 and CD8 T cells in the tumor parenchyma and stroma.

## Materials and methods

### HCC specimens

A total of 86 cases of HCC (61 males and 25 females) were selected from medical records at Koseiren Takaoka Hospital (Toyama, Japan). In each case, HCC was carefully diagnosed as determined by macroscopic and histopathological findings. As a control, corresponding pericancerous non-tumor liver tissues (at least 3 cm away from the tumor site) were also analyzed. None of the individuals had suffered metastasis or had received prior treatment, such as percutaneous ethanol injection, thermal ablation or intra-arterial chemoembolization, which may influence HCC biological behavior, prior to surgery. The HCC samples were classified into four groups according to the International Union Against Cancer tumor-node-metastasis (TNM) classification (21). Pericancerous non-tumor liver tissues were also classified into the following four groups according to the modified histological activity index system (22): Non-chronic hepatitis (NCH), chronic hepatitis (CH), chronic hepatitis with pre-cirrhotic changes (pre-cirrhotic stage, PC) or cirrhosis (C). The detailed profiles of all HCC cases (gender, age, tumor diameter, differentiation, Edmondson staging (23), nodule number, TNM staging, and infiltration into hepatic vein, portal vein or capsule) are shown in Tables I and II. This study was approved by the ethics committee of Koseiren Takaoka Hospital (Toyama, Japan) and written informed consent was obtained from all patients.

### Tissue microarray

Tissue microarrays were constructed as described previously (24). Briefly, in each case, hematoxylin and eosin-stained HCC sections and paired pericancerous liver tissue sections (designated as tumor and peritumor, respectively) were observed under a microscope (Olympus SZX10; Olympus Corporation, Tokyo, Japan). Representative areas of lymphocyte infiltration, away from the necrotic and hemorrhagic areas, were marked and punched with a cylinder (3 mm in diameter) followed by transferal to a recipient block. In total, 172 cores were punched and distributed into 11 recipient blocks. The lesions were placed in duplicate cores adjacent to one another. The blocks were then embedded in paraffin for sectioning at 4 μm.

### Immunohistochemistry

Briefly, following deparaffinization, the sections were subjected to antigen retrieval under microwave heating with target retrieval solution (Dako Cytomation, Kyoto, Japan) for 15 min. Thereafter, the sections were immersed in 0.3% H_2_O_2_ in methanol for 30 min to inhibit endogenous peroxidase activity. The sections were then incubated for 15 min with rabbit anti-human CD4 polyclonal antibodies (1:100; Santa Cruz Biotechnology, Inc., CA, USA) and rabbit anti-human CD8 polyclonal antibodies (1:100; Santa Cruz Biotechnology, Inc.) in phosphate-buffered saline containing 1% normal goat serum (Wako Pure Chemical Industries, Ltd., Tokyo, Japan) and 1% bovine serum albumin (Wako Pure Chemical Industries, Ltd.) under intermittent microwave irradiation, as previously described (25,26). Envision^+^ (Dako Cytomation) for rabbit immunoglobulin was added and the sections were incubated under intermittent microwave irradiation for 15 min. Positive reactions were visualized with 3,3′-diaminobenzidine tetrahydrochloride.

### Morphometrical analysis

CD4- and CD8-positive lymphocytes were classified into the following three groups according to cell distribution: Tumor parenchyma lymphocytes, which were located within a cancer cell nest; tumor stroma lymphocytes, with lymphocytes located in the stroma contacting the cancer cells; and peritumor parenchyma lymphocytes, which were located in the pericancerous liver parenchyma. Morphometrical analysis was performed according to methods described in a previous study (27), for semi-quantitative evaluation of the immunohistochemical findings by two investigators without prior knowledge. Briefly, in each case, using an Olympus SZX10 microscope (Olympus Corporation), 15 independent and intact high power microscopic areas (magnification, ×400) with the most abundant lymphocyte infiltrations were selected (five tumor parenchyma, five tumor stroma and five peritumor parenchyma areas), and the numbers of CD4 and CD8 T cells were counted in each microscopic field. The average numbers of CD4 and CD8 T cells in the five selected microscopic fields signified the CD4 and CD8 expression levels in each HCC or pericancerous liver tissue specimen. For the evaluation of CD4 and CD8 immunoreactions in the tumor stroma, 35 cases were omitted since distinguishing the carcinoma stroma from the surrounding carcinoma parenchyma in these cases was difficult.

### Statistical analysis

The mean and standard error of the mean were calculated for all parameters determined in this study. Statistical analysis was performed using the nonparametric Mann-Whitney U test, one-factor analysis of variance or Spearman’s correlation coefficient by rank test. P<0.05 was considered to indicate a statistically significant difference.

## Results

### Lymphocyte distribution

**Figure 1:**
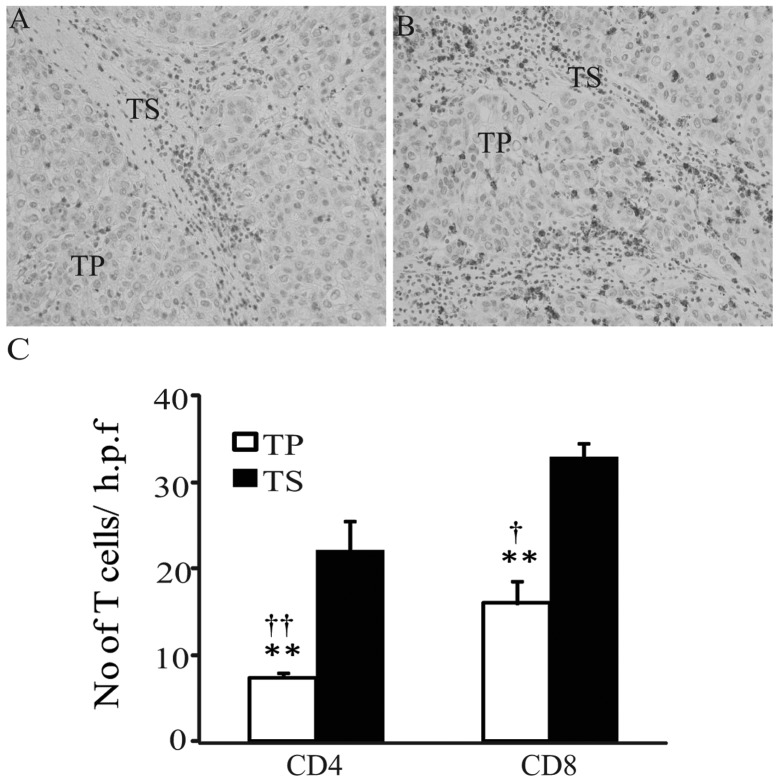
Immunohistochemical analysis of T-cell expression in the TP and TS of hepatocellular carcinoma samples (original magnification, ×400). (A) CD4^+^ and (B) CD8^+^ T-cell expression in TP and TS. (C) Morphometrical analysis was performed. Data are presented as the mean ± standard error of the mean. ^**^P<0.01, TP vs. TS in CD4 and CD8; ^††^P<0.01, CD4 vs. CD8 in TP; ^†^P<0.05, CD4 vs. CD8 in TS. Statistical analyses were performed using the Mann-Whitney U test. TP, tumor parenchyma; TS, tumor stroma; h.p.f., high-power field.

In the HCC samples, CD4 and CD8 T cells were observed in the tumor parenchyma and tumor stroma (Fig. 1A and B), and the intensity of CD4 or CD8 immunoreactivity was homogeneous in all samples examined. The numbers of CD4- and CD8-positive T cells appeared fewer in the tumor parenchyma, compared with those in tumor stroma. In order to semi-quantitatively evaluate the immunohistochemical findings, morphometrical analysis was performed. As shown in Fig. 1C, the average numbers of CD4-and CD8-positive T cells were significantly increased in the tumor stroma, compared with those in the tumor parenchyma (tumor stroma versus tumor parenchyma: CD4, 22±3.6 versus 7.4±0.9; CD8, 32.8±4.2 versus 16±2.5; both P<0.01). Furthermore, the average numbers of CD8-positive T cells in tumor parenchyma and tumor stroma were significantly increased, compared with the numbers of CD4-positive cells (CD8 versus CD4: tumor parenchyma, 16±2.5 versus 7.4±0.9, P<0.01; tumor stroma, 32.8±4.2 versus 22±3.6, P<0.05). This observation suggests that CD8 T cells were predominant in the host anticancer cellular immunity.

### Association between CD4 and CD8 expression and HCC behavior

**Figure 2:**
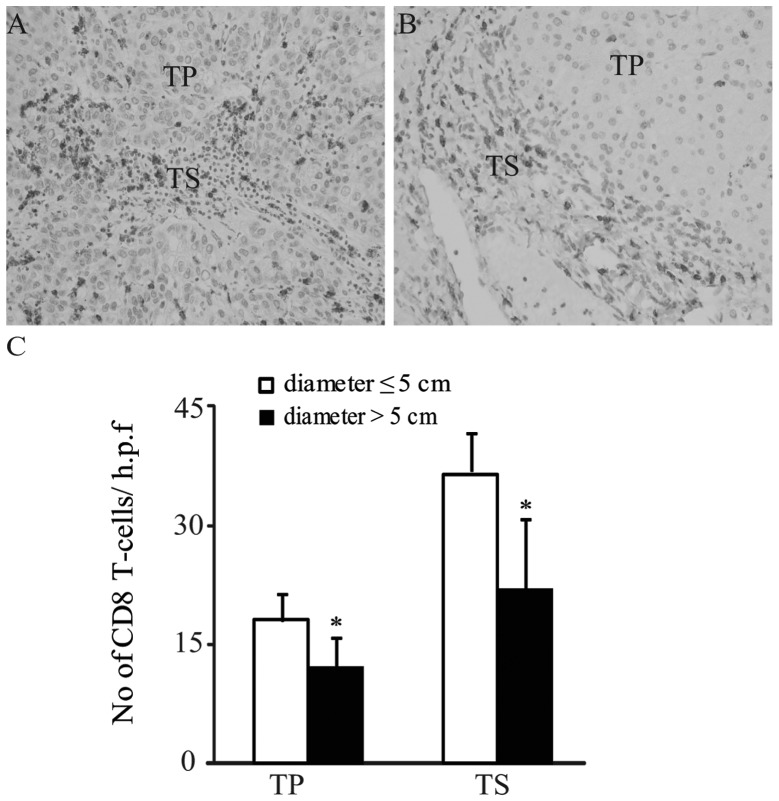
Immunohistochemical analysis of CD8^+^ T-cell expression in the TP and TS of hepatocellular carcinoma samples (original magnification, ×400). CD8^+^ T-cell expression in patients with tumor diameters (A) ≤5 cm and (B) >5 cm. (C) Morphometrical analysis was performed. Data are presented as the mean ± standard error of the mean. ^*^P<0.05, diameter ≤5 cm group vs. diameter >5 cm group in TP and TS. TP, tumor parenchyma; TS, tumor stroma; h.p.f., high-power field.

In the tumor parenchyma and stroma, no significant differences in the CD4 immunoreactions were observed between patients with tumor diameters ≤5 cm and patients with tumor diameters >5 cm (both P>0.05; Tables I and II). By contrast, the average numbers of CD8 T cells in the tumor parenchyma and tumor stroma were significantly increased in patients with tumor diameters ≤5 cm compared with patients with tumor diameters >5 cm (diameter ≤5 cm versus diameter >5 cm: tumor parenchyma, 18.1±3.3 versus 12.2±3.8; tumor stroma, 36.5±4.8 versus 21.9±8.9; both P<0.05; Tables I and II; Fig. 2A–C). Furthermore, in the tumor parenchyma and stroma, no significant differences in either CD4 or CD8 immunoreactivity were detected between age, gender, differentiation, Edmondson staging (23), liver disease background, number of nodules, TNM stage or infiltration into the portal vein, hepatic vein or the capsule variables (Tables I and II). These observations suggest that the numbers of CD8 T cells in HCC parenchyma and stroma may not be correlated with tumor progression or metastasis, but may be correlated with tumor volume.

### Association between CD4 and CD8 expression and background hepatic disease

**Figure 3:**
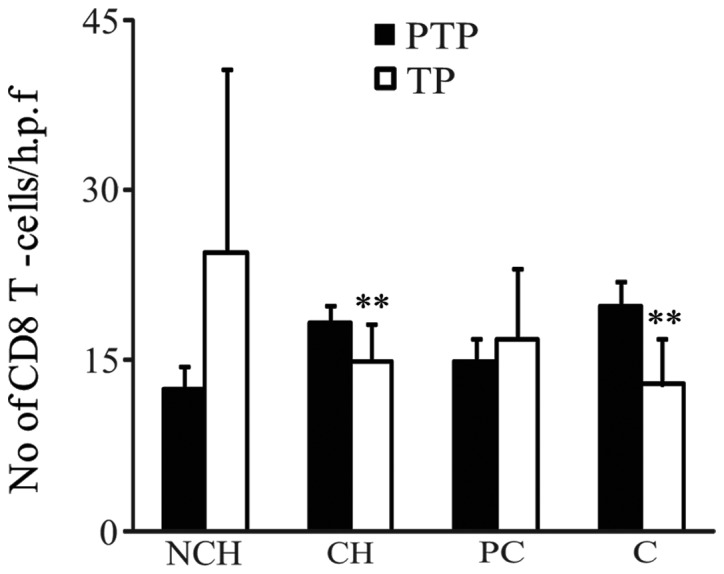
CD8 expression in hepatocellular carcinoma (TP) and paired peritumor liver tissues (PTP). Morphometrical analysis was performed for semi-quantitative evaluation of the immunohistochemical findings. Data are presented as the mean ± standard error of the mean. Statistical analysis was performed using the nonparametric Mann-Whitney U test. ^**^P<0.01, PTP vs. TP in CH and C background groups. NCH, non-chronic hepatitis; CH, chronic hepatitis; PC, pre-cirrhotic stage; C, cirrhosis; PTP, peritumor parenchyma; TP, tumor parenchyma; h.p.f., high-power field.

As shown in Fig. 3, in the CH and C background groups, CD8 expression levels in the peritumor parenchymas were significantly higher than those in the paired tumor parenchymas (peritumor parenchyma vs. tumor parenchyma: CH background, 18.4±1.4 vs. 15.0±3.3; C background, 19.8±2.2 vs. 12.9±4.1; both P<0.01). By contrast, in the NCH and PC background groups, no significant differences in CD8 expression were detected between the tumor parenchyma and peritumor parenchyma (Fig. 3). Furthermore, in HCC and pericancerous liver tissues from all background groups, no significant differences in the CD4 T cells between the tumor parenchyma and peritumor parenchyma (peritumor parenchyma versus tumor parenchyma: NCH background, 4.1±1.4 vs. 6.4±2.8; CH background, 7.9±1.8 vs. 7.1±1.4; PC background, 10.0±3.3 vs. 7.0±1.9; cirrhosis background, 7.6±2.5 vs 8.1±1.8, all P>0.05) were identified.

### Correlation between CD4 and CD8 expression in HCC

**Figure 4:**
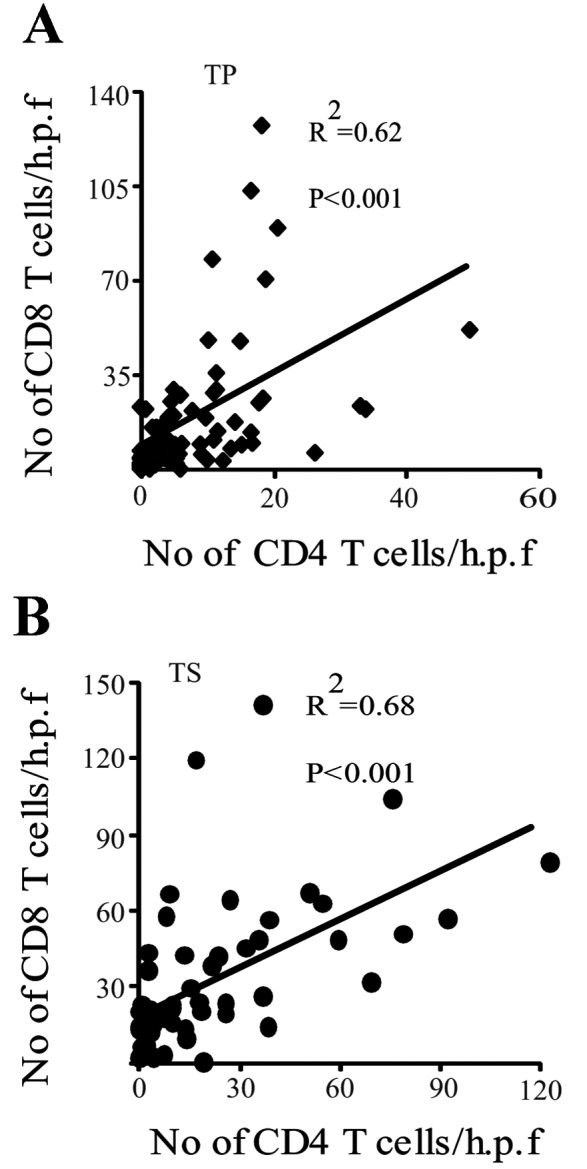
Correlation between CD4 and CD8 expression in (A) TP and (B) TS of HCC samples. Statistical analyses were performed using Spearman’s correlation coefficient by rank test. P<0.001. TP, tumor parenchyma; TS, tumor stroma; HCC, hepatocellular carcinoma; h.p.f., high-power field.

Spearman’s correlation analysis revealed that CD8 expression was positively correlated with CD4 expression in the tumor parenchyma and tumor stroma (correlation coefficient = 0.62 in tumor parenchyma; correlation coefficient = 0.68 in tumor stroma; both P<0.001; Fig. 4A and B).

## Discussion

Solid tumors are composed of parenchyma (neoplastic cells) and stroma. Neoplastic cells are also usually dispersed within the stroma, which is composed of fibroblasts, endothelial cells and a variety of immune cells (28,29). These stromal cells are key in tumor development, tumor control and the response to treatment (9–14). In the present study, the distribution of tumor-infiltrating lymphocytes (TIL) within the tumor parenchyma or tumor stroma was investigated in order to more accurately evaluate the respective impacts of these TILs on the biological behavior of HCC. To the best of our knowledge, no studies have been conducted with regard to the TIL expression in different areas of tumors in association with HCC clinicopathological parameters. The results of the present study revealed significant differences in the intratumoral expression of CD8, but not CD4.

In the present study, a difference in the number of CD8 T cells between the tumor parenchyma and stroma in HCC (tumor parenchyma < tumor stroma) was detected. This may be explained by the evidence from a previous study that tumor microenvironments are rich in immune-cell-derived chemokines (10).

CD8 T cells exert a central role in the immune defense against cancer. For example, CD8-positive CTLs directly contact and kill tumor cells by releasing membrane-lytic granules, such as perforin and granzyme. Indeed, the presence of tumor antigen-specific CD8 T cells has been observed in HCC patients (30). CD8-positive CTLs also kill tumor stroma cells that cross-present antigens. In addition, CTL-derived cytokines, including tumor necrosis factor α, interleukin 4 (IL-4) and IL-10, contribute to tumor rejection by inhibition of tumor stroma formation (20,31–33). In the present study, the average numbers of CD8 T cells in the tumor parenchyma and stroma were higher in patients with tumor diameters ≤5 cm than in patients with tumor diameters >5 cm. In concurrence with this finding, Gao *et al* (16) demonstrated that primary tumor size was inversely correlated with the presence of CD8 T cells in HCC, although no distinction was made regarding the precise location of the T cells. Additionally, in the center (CT) and the invasive margin (IM) of colorectal cancer tumors, CD3, CD8, GZMB (a marker for CD8-positive CTLs) and CD45RO (a marker for memory T cells) expression levels in each tumor region (CT and IM) were negatively correlated with tumor recurrence. High CD8 density, and CD45RO and GZMB expression were correlated with longer overall survival times (11). A study conducted by Chew *et al* (14) further confirmed and complemented these findings; NK and CD8^+^ T cells were observed to be the main proliferating lymphocytes in human HCC. The presence of NK and CD8^+^ T cells was associated with longer survival times, which is concurrent with the finding from another previous study that host anticancer cellular immunity is mainly attributable to CD8-positive CTLs (15). Collectively, these observations suggest that an increased number of CD8 T cells in HCC is associated with longer overall survival times and improved prognosis.

Another finding in the present study was that CD8 expression was significantly increased in the peritumor chronic hepatitis and cirrhotic parenchymas, compared with those in paired tumor parenchymas. This finding is concurrent with the results of a study revealing that the proportion of immune-suppressed regulatory T cells was significantly higher in HCC than that in the non-tumorous liver (34).

The results from the present study demonstrate that CD8-positive T cells are not only important in tumor size control but may also be a valuable prognostic factor. However, the present study did not take account of factors such as survival analysis, phenotypic characterizations (naïve, activated or regulated) and cytotoxic function. Therefore, further studies are required, particularly those that use human HCC specimens with known survival times following HCC resection.

The present study demonstrated that elevated CD8 expression in tumor parenchyma and tumor stroma was correlated with reduced tumor diameter. Therefore, tumor parenchyma and tumor stroma infiltrating CD8 T cells were shown to be involved in HCC diameter control.

## Figures and Tables

**Table I:** Association between the numbers of CD4- and CD8-positive lymphocytes in hepatocellular carcinoma tumor parenchymal tissues, and patient clinicopathological characteristics.

Clinicopathological characteristic	No.	No. CD4 T cells, mean ± SEM	P-value(s)	No. CD8 T cells, mean ± SEM	P-value(s)
Age at diagnosis, years					
≤60	19	7.2±1.7	0.623	15.2±4.3	0.911
>60	67	7.6±1.7		16.5±3.0	
Gender					
Male	61	7.0±0.9	0.137	16.1±2.6	0.061
Female	25	6.4±1.8		15.8±5.9	
Tumor diameter, cm					
≤5	56	7.8±1.3	0.604	18.1±3.3	**0.037**
>5	30	6.2±1.3		12.2±3.8	
Differentiation status					
Well	24	7.2±1.7	0.982	11.0±2.3	0.784
			0.996		0.255
Moderately	39	7.6±1.5	0.982	15.1±2.6	0.784
			0.957		0.507
Poorly	23	6.9±1.2	0.996	22.1±7.7	0.255
			0.957		0.507
Edmondson stage					
I–II	70	7.2±0.9	0.310	16.4±3.3	0.225
III–IV	16	10.5±3.0		14.0±3.6	
Background					
NCH	6	6.4±2.8	0.998	24.5±16.1	0.821
			1.000		0.934
			0.978		0.751
CH	47	7.1±1.4	0.998	15.0±3.3	0.821
			0.999		0.996
			0.980		0.989
PC	11	7.0±1.9	1.000	16.9±6.2	0.934
			0.999		0.996
			0.991		0.973
C	22	8.1±1.8	0.978	12.9±4.1	0.751
			0.980		0.989
			0.991		0.973
Nodule number					
Single	77	7.4±0.9	0.400	16.7±2.7	0.667
Double	9	6.6±2.8		9.9±3.0	
Portal vein infiltration					
Yes	17	6.2±2.6	0.667	21.7±7.8	0.633
No	69	7.3±0.9		14.5±2.4	
Hepatic vein invasion					
Yes	16	7.2±1.9	0.709	13.8±4.1	0.228
No	70	7.4±1.0		16.7±3.0	
Infiltration into capsule					
Yes	29	7.4±1.4	0.608	17.7±5.2	0.812
No	57	7.2±1.1		15.1±2.7	
TNM stage					
I–II	54	7.7±1.2	0.364	16.3±3.1	0.301
III–IV	32	6.0±1.1		13.2±3.7	

P-values were obtained with nonparametric Mann-Whitney U test or one-factor analysis of variance. Bold P-value denotes statistical significance. SEM, standard error of the mean; NCH, non-chronic hepatitis; CH, chronic hepatitis; PC, chronic hepatitis with cirrhotic changes; C, cirrhosis.

**Table II:** Association between the numbers of CD4- and CD8-positive lymphocytes in hepatocellular carcinoma tumor stromal tissues, and patient clinicopathological characteristics.

Clinicopathological characteristic	No.	CD4 T cells, n, mean ± SEM	P-value(s)	CD8 T cells, n, mean ± SEM	P-value(s)
Age at diagnosis, years					
≤60	12	23.9±10.6	0.400	33.3±10.0	0.842
>60	39	21.8±3.8		33.1±4.8	
Gender					
Male	37	24.0±4.6	0.315	31.3±4.1	0.849
Female	14	17.2±5.9		37.8±11.5	
Tumor diameter, cm					
≤5	40	23.1±4.4	0.519	36.5±4.8	**0.022**
>5	11	19.4±7.2		21.9±8.9	
Differentiation status					
Well	10	20.7±6.8	0.911	28.3±5.9	0.870
			0.971		0.886
Moderately	27	24.7±6.0	0.911	34.0±6.1	0.870
			0.757		0.999
Poorly	14	18.1±5.1	0.971	34.3±9.5	0.886
			0.757		0.999
Edmondson stage					
I–II	31	20.7±4.0	0.317	32.7±4.4	0.858
III–IV	20	27.4±8.6		36.7±11.5	
Background					
NCH	3	8.8±5.5	0.894	5.3±3.9	0.530
			0.154		0.149
			0.946		0.665
CH	33	20.9±4.4	0.894	32.1±5.5	0.530
			0.117		0.416
			0.997		0.996
PC	4	54.9±25.3	0.154	59.0±19.9	0.149
			0.117		0.416
			0.141		0.424
C	11	18.9±5.7	0.946	29.7±6.8	0.665
			0.997		0.996
			0.141		0.424
Nodule number					
Single	43	22.2±4.1	0.728	32.7±4.8	0.368
Double	8	21.6±6.9		36.9±7.6	
Portal vein infiltration					
Yes	9	24.1±8.0	0.652	43.2±16.4	0.942
No	42	22.0±4.1		31.3±3.8	
Hepatic vein invasion					
Yes	15	22.5±6.2	0.879	42.0±10.5	0.558
No	36	22.5±4.6		31.2±4.1	
Infiltration into capsule					
Yes	18	21.9±5.0	0.573	38.2±9.0	0.700
No	33	22.3±4.9		29.0±3.5	
TNM stage					
I–II	31	23.9±5.3	0.978	31.8±4.9	0.867
III–IV	20	20.3±4.7		34.7±7.7	

P-values were obtained with nonparametric Mann-Whitney U test or one-factor analysis of variance. Bold P-value denotes statistical significance. SEM, standard error of the mean; NCH, non-chronic hepatitis; CH, chronic hepatitis; PC, chronic hepatitis with cirrhotic changes; C, cirrhosis.
